# Phase I Trial of Regorafenib, Hydroxychloroquine, and Entinostat in Metastatic Colorectal Cancer

**DOI:** 10.1093/oncolo/oyac078

**Published:** 2022-05-13

**Authors:** Thomas B Karasic, Timothy J Brown, Charles Schneider, Ursina R Teitelbaum, Kim A Reiss, Tara C Mitchell, Ryan C Massa, Mark H O’Hara, Lisa DiCicco, Luis Garcia-Marcano, Ravi K Amaravadi, Peter J O’Dwyer

**Affiliations:** Abramson Cancer Center, University of Pennsylvania, Philadelphia, PA, USA; Abramson Cancer Center, University of Pennsylvania, Philadelphia, PA, USA; Abramson Cancer Center, University of Pennsylvania, Philadelphia, PA, USA; Abramson Cancer Center, University of Pennsylvania, Philadelphia, PA, USA; Abramson Cancer Center, University of Pennsylvania, Philadelphia, PA, USA; Abramson Cancer Center, University of Pennsylvania, Philadelphia, PA, USA; Abramson Cancer Center, University of Pennsylvania, Philadelphia, PA, USA; Abramson Cancer Center, University of Pennsylvania, Philadelphia, PA, USA; Abramson Cancer Center, University of Pennsylvania, Philadelphia, PA, USA; Abramson Cancer Center, University of Pennsylvania, Philadelphia, PA, USA; Abramson Cancer Center, University of Pennsylvania, Philadelphia, PA, USA; Abramson Cancer Center, University of Pennsylvania, Philadelphia, PA, USA

**Keywords:** colorectal cancer, autophagy, antiangiogenesis, epigenetics, phase I

## Abstract

**Background:**

The antiangiogenic tyrosine kinase inhibitor regorafenib provides a survival benefit in patients with previously treated metastatic colorectal cancer (CRC). Antiangiogenic therapy causes hypoxic stress within tumor cells, which activates autophagy as a survival mechanism. The histone deacetylase inhibitor (HDAC) entinostat increases dependence on autophagy through epigenetic mechanisms. Hydroxychloroquine (HCQ) blocks autophagy by blunting lysosomal acidification. We hypothesized that HCQ and entinostat would be tolerable with regorafenib and potentiate the antitumor response.

**Methods:**

This was a 3+3 phase I trial of HCQ and entinostat with regorafenib in patients with metastatic CRC. The primary objective was safety, and the secondary objective was clinical efficacy.

**Results:**

Twenty patients received study therapy. Six evaluable patients were enrolled at each of the three planned dose levels, one patient at an intermediate dose level, and one additional patient withdrew consent after 4 days to receive treatment closer to home. One dose-limiting toxicity was noted in the study at dose level 2 (grade 3 fatigue). Seven patients discontinued therapy due to related toxicities; rapid weight loss was near universal, with a median weight loss of 4.4 kg (range 1.5-12.2 kg) in the first 2 weeks of treatment. No objective responses were observed.

**Conclusion:**

The combination of regorafenib, HCQ, and entinostat was poorly tolerated without evident activity in metastatic CRC.

**ClinicalTrials.gov Identifier:**

NCT03215264

## Teaser

The antiangiogenic tyrosine kinase inhibitor regorafenib provides a survival benefit in patients with previously treated metastatic colorectal cancer (CRC). This article reports results of a trial that investigated whether hydroxychloroquine and entinostat would be tolerable with regorafenib and potentiate the antitumor response.

**Lessons Learned**

- Regorafenib, entinostat, and hydroxychloroquine resulted in rapid weight loss and excess fatigue in the majority of patients.
- No antitumor efficacy was observed among 20 patients.

## Discussion

**Figure 1.**
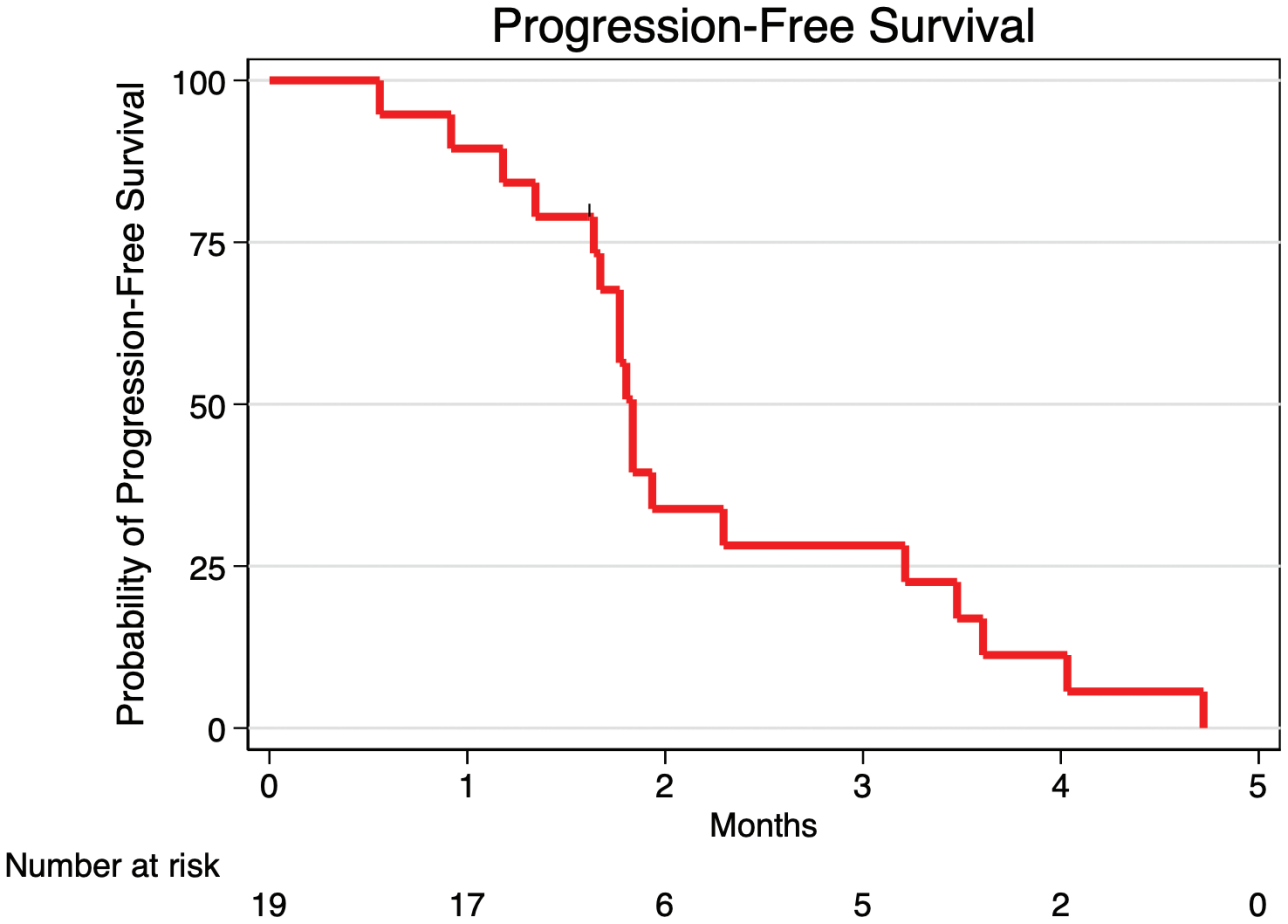
Kaplan-Meier plot of progression-free survival.

Angiogenesis is a hallmark of cancer and targeting angiogenesis has been a therapeutic strategy in advanced colorectal cancer since the approval of bevacizumab.^1^ Regorafenib, an antiangiogenic oral tyrosine kinase inhibitor (TKI) that inhibits the vascular endothelial growth factor receptor was approved in chemotherapy-refractory patients on the basis of improved overall survival versus placebo.^2^ However, median progression-free survival in patients receiving regorafenib was less than 2 months, and the objective response rate was only 1%, indicating a need for more effective therapies in this population (Figure 1).

We have previously demonstrated that antiangiogenic therapy results in tumor cell death through hypoxia and that cancer cells can survive through autophagy, a metabolic program to break down intracellular components for energy.^3^ The HDAC inhibitor entinostat further increases dependence on autophagy while HCQ effectively blocks autophagy by preventing acidification of the lysosome, the final step in the pathway.^4,5^ Our preclinical modeling in colon cancer cell lines demonstrated synergy between the autophagy inhibitor chloroquine and the HDAC inhibitor vorinostat under hypoxic conditions [O’Dwyer P, unpublished data], and we designed a phase I trial to determine if HCQ with entinostat combined with regorafenib would be tolerable and effective in refractory advanced CRC.

Twenty patients received study therapy. While only one dose-limiting toxicity (DLT) was observed and the study reached the maximum planned dose, the combination was poorly tolerated and unacceptable toxicity was common. Seven patients discontinued therapy due to therapy-related toxicities. Weight loss was rapid and pronounced and affected every patient beginning with the first toxicity assessment. The median weight loss after 2 weeks on therapy was 4.4 kg (range 1.5-12.2 kg) and was 3.6 kg after completing cycle 1 (range 1.5-14.4 kg). Fatigue and anorexia were also common, each occurring in 10 patients. A single DLT was observed at dose level 2 (grade 3 fatigue). There was no evident anti-cancer activity, with a median progression-free survival of 1.8 months and a median overall survival of 5.2 months. No objective responses were observed and no patient remained on study therapy beyond 4 cycles. Further investigation of this combination is not warranted in patients with CRC.

## Trial Information

**Table:** 

Disease	Colorectal cancer
Stage of disease/treatment	Metastatic
Prior therapy	Fluoropyrimidine, oxaliplatin, irinotecan
Type of study	Phase I
Primary endpoint	Recommended phase II dose (RP2D)
Secondary endpoints	Objective response rate, toxicity rates by category, overall survival, progression-free survival, duration of response
Investigator’s assessment	Poorly tolerated/not feasible

### Additional Details of Endpoints or Study Design

This was a standard 3+3 phase I trial. DLTs were assessed in the first 4 weeks. Non-hematologic toxicities of grade 3 or higher were considered DLTs except for rash attributable to regorafenib or nausea/vomiting or diarrhea that improved to grade 1 within 72 hours. Hematologic DLTs were defined as febrile neutropenia, grade 4 neutropenia lasting more than 7 days, a platelet count of less than 25 000, or a platelet count of less than 50 000 with bleeding. Patients were evaluable for toxicity if they took at least one dose of any study drug and were evaluable for efficacy if they completed at least 4 weeks of therapy and underwent repeat imaging. A planned expansion at the RP2D was not pursued due to excess toxicity.

Table 1 shows dose escalation. Table 2 details adverse events.

**Table 1.** Dose escalation table.

Dose level	Dose hydroxychloroquine (mg/day)	Schedule of HCQ administration	Dose entinostat
−1	400 mg	200 mg q12h	2 mg weekly
1	600 mg	200 mg qAM/ 400 mg qPM	3 mg weekly
2	600 mg	200 mg qAM/ 400 mg qPM	5 mg weekly
2A	800 mg	400mg qAM/400mg qPM	5 mg weekly
2B	1000 mg	400mg qAM/600mg qPM	5 mg weekly
3	1200 mg	600 mg q12h	5 mg weekly

**Table 2.** Adverse events.

Adverse event	G1	G2	G3	G4	Total
Weight loss	11	1			12
Fatigue	1	7	2		10
Anorexia	8	2			10
Alkaline phosphatase increased	7		2		9
Platelet count decreased	6	1	1		8
White blood cell decreased	5	1	1		7
Nausea	3	4			7
Anemia	2	3	1		6
Palmar-plantar erythrodysesthesia syndrome	2	2	1		5
Diarrhea	2	2			4
Aspartate aminotransferase increased	3				3
Vomiting	2	1			3
Mucositis oral	1	2			3
Rash maculo-papular	1	1	1		3
Blood bilirubin increased	1	1			2
Neutrophil count decreased	1	1			2
Pruritus	1	1			2
Mucosal infection	1	1			2
Dyspnea	1	1			2
Hoarseness	2				2
Thyroid stimulating hormone increased	1				1
Lymphocyte count decreased			1		1
Alanine aminotransferase increased	1				1
Pain	1				1
Fever		1			1
Gait disturbance	1				1
Bloating	1				1
Flatulence	1				1
Abdominal pain		1			1
Rectal hemorrhage		1			1
Dry skin			1		1
Rash acneiform			1		1
Headache	1				1
Dysgeusia	1				1
Hypophosphatemia		1			1
Papulopustular rash			1		1
Lung infection		1			1
Cough	1				1
Arthralgia		1			1
Muscle weakness lower limb		1			1
Hypotension		1			1
Urinary frequency	1				1

## Drug Information

**Table:** 

	Drug 1	Drug 2	Drug 3
Generic/working name	Regorafenib	Hydroxychloroquine	Entinostat
Company name drug type	Stivarga		
Drug class	Small molecule	Autophagy inhibitor	Histone deacetylase inhibitor
Dose	Antiangiogenic tyrosine kinase inhibitor	400-1200	2-5
Unit	80-160 mg	Mg	Mg
Route	Oral	Oral	Oral
Schedule of administration	Daily for 21 days of each 28-day cycle	In 2 divided doses daily	Daily

## Patient Characteristics

**Table:** 

Number of patients, male	**10**
Number of patients, female	**10**
Stage	IV
Age: median (range)	61 (37-81) years
Number of prior systemic therapies: median(range)	**3 (2-8)**
Performance status: ECOG	0: 8 1: 12 2: 0 3: 0 4: 0
Cancer types or histologic subtypes	Colorectal cancer, 20

## Primary Assessment Method

**Table:** 

Title	**Efficacy**	
Number of patients screened	**27**	
Number of patients enrolled	20	
Number of patients evaluable for toxicity	20	
Number of patients evaluated for efficacy	**19**	
Evaluation method	RECIST 1.1	
**Response assessment**	***N***	**%**
CR	0	0
PR	0	0
SD	6	31.6
PD	13	68.4
**(Median) duration assessments**		
PFS	1.8 months	CI: 1.3-2.3
OS	5.2 months	CI: 2.9-7.5
Outcome notes	Patients were assessed every 2 weeks for toxicity. Restaging imaging evaluations were conducted at 8 weeks, 16 weeks, and then every 8-12 weeks.	

## Assessment, Analysis, and Discussion

**Table:** 

Completion	Study terminated prior to completion
Investigator’s assessment	Poorly tolerated/not feasible

Antiangiogenic therapies modestly improve overall survival in CRC when combined with first- and second-line chemotherapies, and the TKI regorafenib offers a survival benefit in chemotherapy-refractory patients. Enhancing antiangiogenic therapy in CRC has proven elusive in a variety of studies.^6^ We have previously demonstrated an increased response rate with HCQ combined with 5-fluorouracil, oxaliplatin (FOLFOX), and bevacizumab in untreated advanced CRC.^7^ However, no improvement was seen in overall survival compared to historical controls, nor was it possible to separate out whether HCQ might be interacting with the chemotherapy or with bevacizumab, as both induce autophagy.

Regorafenib is the only antiangiogenic therapy used as a single agent in CRC, and we designed this study to test our hypothesis that autophagy inhibition would enhance antiangiogenic therapy in CRC, and that the HDAC inhibitor entinostat would further promote autophagy dependence. The combination of biweekly entinostat with the highly similar TKI sorafenib showed no toxicities at full doses beyond those expected with each agent individually, and we have demonstrated little additional toxicity with the addition of HCQ to a variety of cancer regimens.^7-11^ The HDAC inhibitor vorinostat combined with HCQ is also well-tolerated in CRC.^12,13^

**Figure 2.**
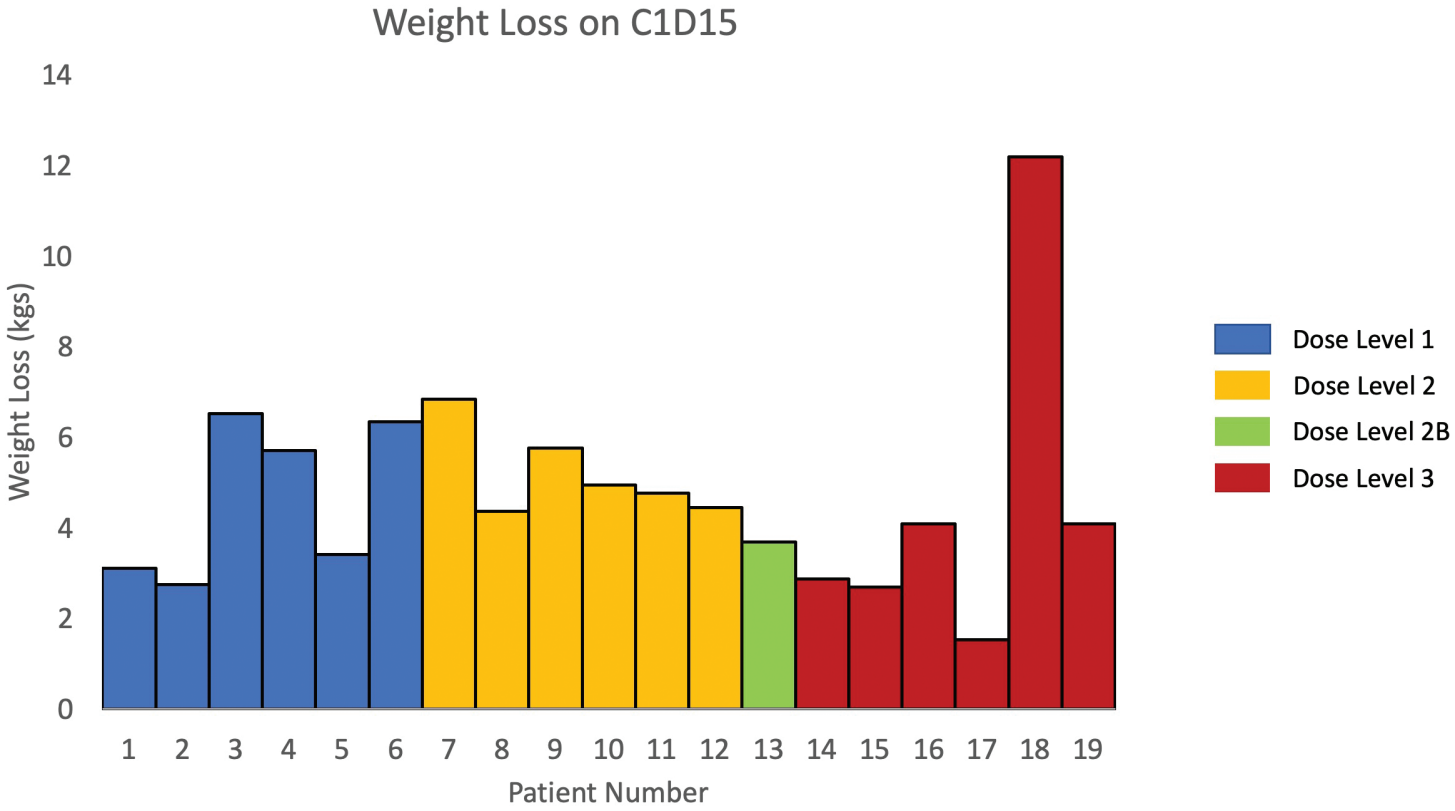
Graph of weight loss by dose level for each patient.

Nevertheless, this triplet combination was poorly tolerated, with rapid weight loss in nearly every patient along with early onset fatigue and anorexia (Figure 2). Weight loss primarily occurred during the first two weeks of therapy and then stabilized but did not reverse. To mitigate toxicity, the initial regorafenib dose was lowered per the ReDOS study to 80mg after the first five patients, but toxicity persisted at similar rates, and most patients were unable to escalate to full dose regorafenib.^14^ No dose dependency of weight loss or any other toxicity was clearly observed. The TKI sunitinib was previously found to have a interaction with HCQ, with accumulation of active sunitinib metabolites resulting in excess toxicity.^15^ We did not perform pharmacokinetic testing and cannot exclude the possibility of a drug-drug interaction, although the lack of a relationship between dosing and toxicity makes this less likely. We hypothesize instead that a metabolic interaction may underlie the toxicities observed, though it is unclear if this could be a class effect or specific to these agents, and further metabolic analyses are planned.

While expansion to 20 patients at the recommended phase II dose was not pursued as initially planned due to the toxicity observed, efficacy analysis of the patients treated on the study shows median PFS (1.8 months) and OS (5.2 months) that are similar to those seen with regorafenib alone, and no objective responses. Translating epigenetic therapies into solid tumors remains challenging, with the noteworthy recent failures of entinostat with hormone therapy in breast cancer and immunotherapy in lung cancer.^16,17^ Newer combinations with immunotherapy and antiangiogenic therapies plus HCQ may instead hold greater promise to improve treatment for genomically defined subsets of advanced CRC.^18^

In conclusion, the combination of regorafenib, entinostat, and HCQ was poorly tolerated and had efficacy similar to regorafenib alone. Further investigation of this combination is not warranted.

## Data Availability

The data underlying this article are available in the article and in its online supplementary material.
